# The structure of nontypeable *Haemophilus influenzae* SapA in a closed conformation reveals a constricted ligand-binding cavity and a novel RNA binding motif

**DOI:** 10.1371/journal.pone.0256070

**Published:** 2021-10-15

**Authors:** Petra Lukacik, C. David Owen, Gemma Harris, Jani Reddy Bolla, Sarah Picaud, Irfan Alibay, Joanne E. Nettleship, Louise E. Bird, Raymond J. Owens, Philip C. Biggin, Panagis Filippakopoulos, Carol V. Robinson, Martin A. Walsh

**Affiliations:** 1 Diamond Light Source, Harwell Science & Innovation Campus, Didcot, Oxfordshire, United Kingdom; 2 Research Complex at Harwell, Harwell Science and Innovation Campus, Didcot, Oxfordshire, United Kingdom; 3 Department of Chemistry, University of Oxford, Oxford, United Kingdom; 4 Structural Genomics Consortium, University of Oxford, Oxford, United Kingdom; 5 Department of Biochemistry, University of Oxford, Oxford, United Kingdom; 6 Wellcome Trust Centre for Human Genetics, University of Oxford, Oxford, United Kingdom; National Institute for Medical Research, Medical Research Council, London, UNITED KINGDOM

## Abstract

Nontypeable *Haemophilus influenzae* (NT*Hi*) is a significant pathogen in respiratory disease and otitis media. Important for NT*Hi* survival, colonization and persistence *in vivo* is the Sap (sensitivity to antimicrobial peptides) ABC transporter system. Current models propose a direct role for Sap in heme and antimicrobial peptide (AMP) transport. Here, the crystal structure of SapA, the periplasmic component of Sap, in a closed, ligand bound conformation, is presented. Phylogenetic and cavity volume analysis predicts that the small, hydrophobic SapA central ligand binding cavity is most likely occupied by a hydrophobic di- or tri- peptide. The cavity is of insufficient volume to accommodate heme or folded AMPs. Crystal structures of SapA have identified surface interactions with heme and dsRNA. Heme binds SapA weakly (K_d_ 282 μM) through a surface exposed histidine, while the dsRNA is coordinated via residues which constitute part of a conserved motif (estimated K_d_ 4.4 μM). The RNA affinity falls within the range observed for characterized RNA/protein complexes. Overall, we describe in molecular-detail the interactions of SapA with heme and dsRNA and propose a role for SapA in the transport of di- or tri-peptides.

## Introduction

Respiratory illness currently ranks as the third leading cause of death globally [1], with rates peaking for the very young and the elderly [2, 3]. High incidence of the gram-negative bacterium nontypeable *Haemophilus influenzae* (NT*Hi*) in these illnesses makes it a strategic target for biochemical investigation. Following triggers that are not yet fully understood, NT*Hi* can transition from a commensal into a pathogen, leading to opportunistic respiratory tract infections such as in chronic obstructive pulmonary disease (COPD) [4], pneumonia and exacerbations of cystic fibrosis. Outside of the respiratory context, NT*Hi* is also a major pathogen in meningitis and otitis media (OM), with the latter being a leading cause of specifically treated disease in children both in the UK and worldwide [5, 6]. Taken together the costs of NT*Hi* related diseases represent a major economic and social burden.

While antibiotic resistance in NT*Hi* is primarily effected through β-lactamase, the bacterium is also gaining resistance to other antibiotic classes [7, 8]. Even without this additional threat, ampicillin resistant strains now exceed 30% in some countries, which has inevitably led to higher rates of treatment failure, increased costs, and decreased availability for severely or chronically ill patients. The extracellular capsule targeted by the now routine *Hi* type b (Hib) vaccine [9] is absent in NT*Hi* and, as yet, no effective vaccine exists against it. Furthermore, with the introduction of the Hib vaccine, NT*Hi* has become more prevalent [10]. NT*Hi* vaccine development has proven to be challenging, in part due to high heterogeneity of outer membrane protein vaccine candidates and antigenic drift present in patients with long-term NT*Hi* infection [11, 12]. The development of novel treatments, antimicrobials and vaccines is therefore much needed and would be aided by an improved understanding of NT*Hi* pathogenic survival strategies.

An essential system for NT*Hi* survival and persistence *in vivo* is the Sap (sensitivity to antimicrobial peptides) transporter. This multi-component system uses a periplasmic protein (SapA) to bind and deliver substrates to the heterodimeric inner membrane-associated Sap permease (SapBC). The Sap ABC transporter is completed by two membrane-associated nucleotide-binding proteins (SapD & SapF) that hydrolyze ATP to provide energy for substrate transport across the bacterial inner membrane.

Studies based mainly on NT*Hi sap* mutants have shown a decrease in sensitivity to host antimicrobial peptides (AMPs) both *in vitro* and *in vivo*. As part of the innate immune system, AMPs contribute to the first line of defense against infection. These small (<10 kDa), cationic or amphipathic peptides have diverse structures and mostly act by disrupting cell membranes leading to cell lysis and death. Cell killing assays that tested *sapA*, *sapD*, and *sapBC* mutant strains have shown these to be more sensitive to AMP-mediated killing. Specifically, the *sapA* mutant is more sensitive to chinchilla Beta Defensin 1 (cBD1) [13]; *sapD* to cBD1 [14], cathelicidin LL-37 and human Beta Defensin 3(hBD3) [15]; and *sapBC* to LL-37 and hBD3 [16]. Growth of *sapA* [13], *sapD* [14] and *sapF* [15] mutant strains was attenuated in a chinchilla model of OM. The *sap* genes have also been postulated to have a role in iron acquisition [17, 18], with s*apA*, *sapBC* and *sapF* mutants demonstrating an inability to utilize heme for growth in specific nutrient controlled media. Evidence has also been presented for a direct interaction of SapA protein with heme [17, 19], and with cBD1 and LL-37 [14]. Additionally, hBD1, hBD2, hBD3, LL-37, human neutrophil protein 1, and melittin were shown to displace bound heme from SapA [17]. NT*Hi* [20] and *K*. *pneumoniae* [21] *sapA* mutants have decreased adhesion to epithelial cells and increased invasiveness, while the *sapF* mutant has altered biofilm morphology [15]. The importance of the *sap* genes in resistance to AMPs has been demonstrated in other bacterial pathogens, including *H*. *ducreyi* [22, 23], *S*. *typhimurium* [24–27] and *E*. *chrysanthemi* [28].

Based on these studies a model has been proposed [17] for a multifunctional Sap transporter where SapA shuttles heme and AMPs to the Sap ABC transporter which moves them across the cytoplasmic membrane. Within the cytoplasm, AMPs are degraded and heme is utilized in a nutritional context. The disruption of the *sap* genes clearly has dramatic effects but the precise molecular interactions that are responsible for these effects are poorly understood. To shed light on these issues we have determined the crystal structure of NT*Hi* SapA and characterized its interactions with a number of chemically distinct ligands.

## Results

### SapA crystallized in open and closed conformations

**Fig 1:**
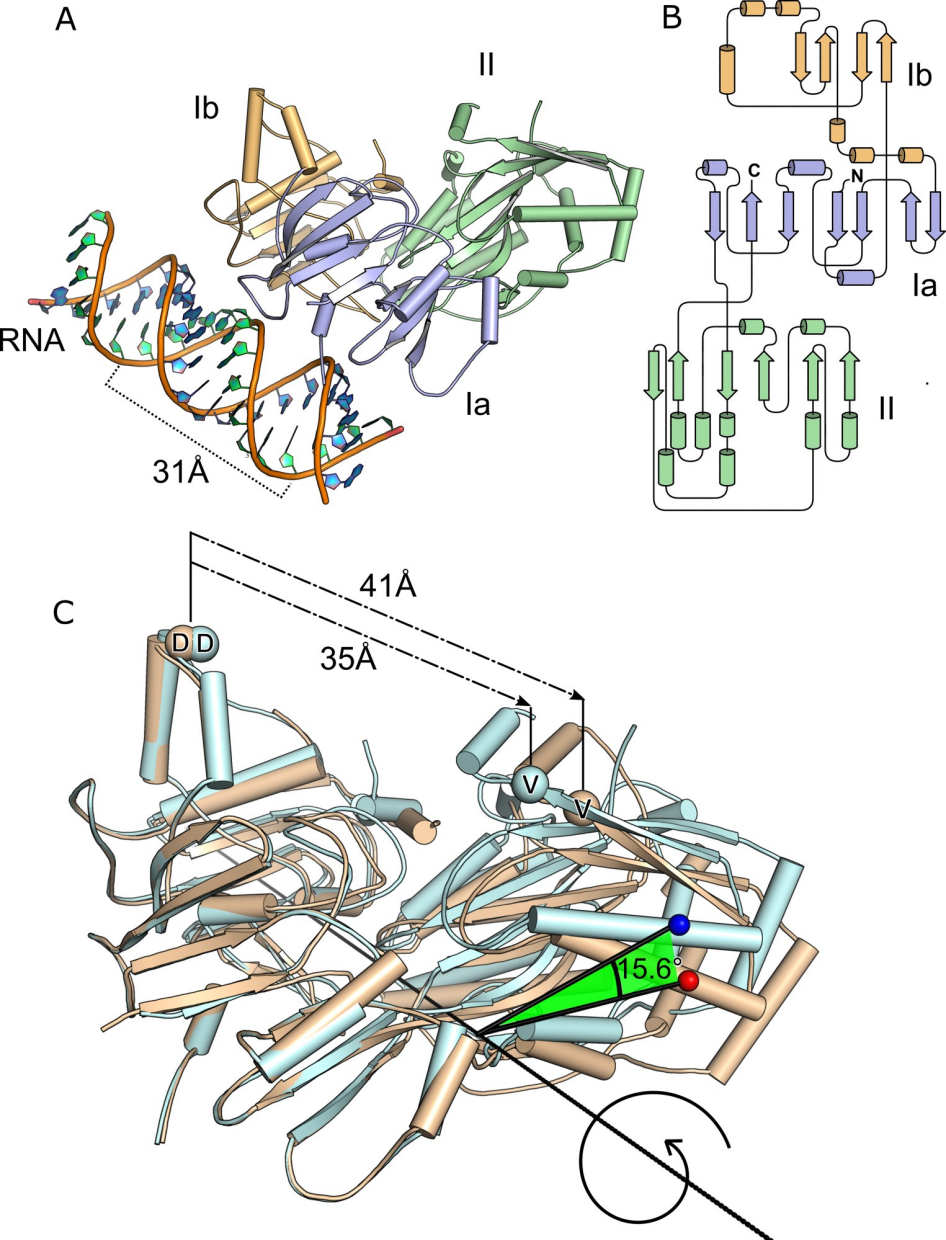
Overall SapA structure. A, the overall structure of SapA_closed_ coloured by domain. Each SapA monomer was co-crystallised with a single-stranded A-form RNA molecule. The antisense strand (turquoise rings) completing the duplex is contributed by a symmetry mate SapA_closed_ molecule not shown in the diagram. The pitch distance of the RNA helix is indicated. B, topology diagram of SapA. C, comparison of the closed (light cyan) and open (wheat) conformations of SapA. Rotation angle around axis was calculated and visualised with the program *ProSMART*. The distances between the Cα atoms of residues Asp 222 and Valine 435 are indicated in the closed and open conformations.

Despite numerous optimization attempts, the expression of SapA was poor, with a final yield of ≈5 mg pure protein per 50 g of *E*.*coli* cells. Crystals were nevertheless obtained and the structure of the 60 kDa SapA protein was solved to a resolution of 2.6 Å. The structure confirmed the classification of SapA into the Substrate Binding Protein (SBP) superfamily, as was predicted from sequence analysis. SBPs are a class of proteins that are often associated with membrane protein complexes, in particular, ABC transporters [29]. Within the SBP superfamily, SapA can be further categorized as a Class II SBP [30] based on β-sheet topology, and to SBP Cluster C by the atypical presence of an additional domain of approximately 15 kDa [29, 31]. In the SapA crystal structure this additional domain, Ib, associates with domain Ia to form one lobe, whilst domain II forms the second lobe (Fig 1A). Domain Ia encompasses residues 35:68, 230:312, 532:561; residues 69:229 constitute domain Ib, and domain II is formed by residues 313:531. All three domains present a mixed α/β architecture with central β-sheets surrounded by α-helices (Fig 1B). The polypeptide chain is for the most part in well-defined electron density, except for residues 139–162 which form a loop that could not be modeled.

**Fig 2:**
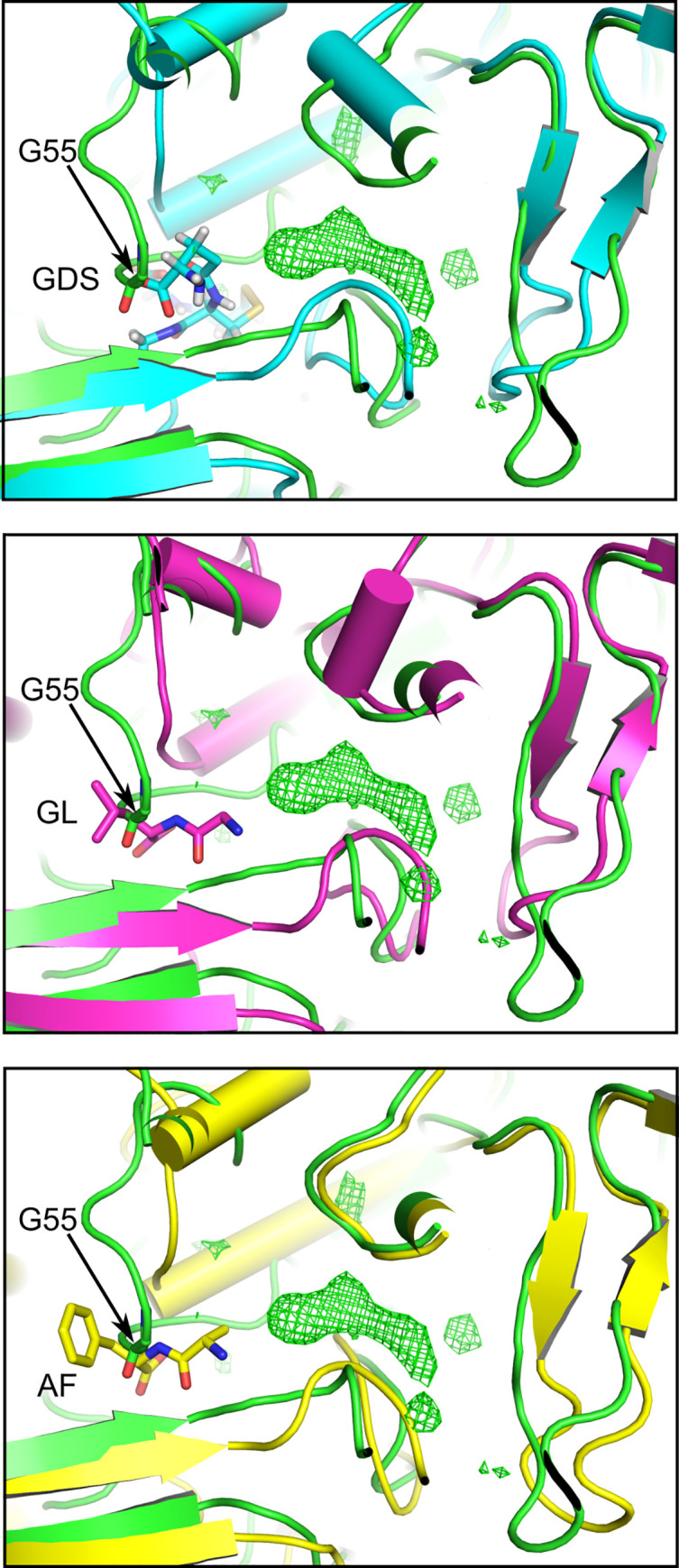
Superimposition of the NT*Hi* SapA_closed_ structure (green) with its closest structural homologs. A, *Hp*HbpA (cyan, PDB = 3M8U); B, *Ec*DppA (magenta, PDB = 1DPP); C, *Ps*DppA (yellow, PDB = 4QFL). The dipeptide (GL,AF) and oxidised glutathione disulphide (GDS) ligands are shown in stick representation. The green mesh represents the difference density *Fo-Fc* within the ligand-binding cavity of NT*Hi* SapA_closed_ (contoured at 3σ).

We crystallized SapA in several forms (Table 1, S1A–S1C Fig): the first, denoted as SapA_closed_ (PDB = 7OFZ), contains a single SapA chain with its two lobes in close contact with each other. The second, denoted as SapA_mixed_ (PDB = 7OG0), contains two copies of SapA, with chain A adopting a similar conformation to SapA_closed_ and chain B showing substantially greater separation between the lobes, this conformation is denoted SapA_open_. As a comparison, the distance between Asp 222 and Val 435, residues that respectively reside near to the termini of lobes I and II, are 35 Å apart in SapA_closed_ and over 40 Å apart in SapA_open_. A transition from the open to closed state requires a 15.6° rotation around the axis bisecting domains I and II (Fig 1C). Conformational shifts such as these are characteristic of the SBP superfamily upon binding/release of their cognate ligand [29] and have been described as a “Venus flytrap” mechanism [32]. The structure of SapA_closed_ aligns well with the structures of its homologs in their ligand-occupied states (Fig 2). Specifically, the alignment rmsds of SapA_closed_ with ligand occupied *H*. *parasuis* HbpA (*Hp*HbpA), *E*. *coli* DppA (*Ec*DppA) and *Pseudoaltermonas* sp. SM9913 DppA (*Ps*DppA) are 1.71 Å, 2.33 Å and 2.27 Å, respectively. The observation that the SapA_closed_ structure was in the same conformation as its ligand-bound homologs was unexpected as no extraneous ligands were added to the purified protein or crystals.

**Table 1:** Crystallographic data collection and refinement statistics.

Crystal form	SapA_closed_	SapA_mixed_	SapA_heme_
**PDB ID**	7OFZ	7OG0	7OFW
**Data Collection**			
Wavelength (Å)	0.9200	0.9174	0.9763
Resolution range (Å)^a^	72.2–2.62 (2.71–2.62)	65.74–2.61 (2.70–2.61)	71.36–3.15 (3.26–3.15)
Space group	P 4 21 2	P 21 21 2	P 4 21 2
Unit cell (Å,°)	144.4 144.4 62.0 90 90 90	143.3 148.0 59.6 90 90 90	142.7 142.7 60.6 90 90 90
Total reflections^a^	38352 (3840)	144902 (14854)	116349 (11497)
Unique reflections^a^	19270 (1929)	38143 (3848)	11330 (1111)
Multiplicity^a^	2.0 (2.0)	3.8 (3.9)	10.3 (10.3)
Completeness (%)^a^	94.9 (96.9)	96.9 (98.7)	99.9 (99.8)
Mean I/σI^a^	18.2 (1.1)	11.2 (2.1)	16.9 (3.3)
R-merge^a^	0.031 (0.600)	0.089 (0.623)	0.128 (1.029)
R-meas^a^	0.044 (0.848)	0.104 (0.721)	0.135 (1.084)
CC1/2^a^	0.999 (0.571)	0.997 (0.698)	0.998 (0.845)
**Refinement**			
Reflections used in refinement^a^	19267 (1929)	38142 (3848)	11326 (1110)
R-work^a^	0.218 (0.318)	0.199 (0.286)	0.239 (0.341)
R-free^a^	0.263 (0.381)	0.249 (0.308)	0.276 (0.366)
Number of non-hydrogen atoms	4527	8917	4395
Protein	4005	8030	3963
RNA/ions	407	728	387
heme	-	-	43
solvent	114	157	2
RMSD bond lengths (Å)	0.014	0.014	0.01
RMSD bond angles (°)	1.8	1.4	1.5
Ramachandran favored (%)	96.1	97.3	96.5
Ramachandran allowed (%)	3.7	2.0	3.1
Ramachandran outliers (%)	0.2	0.7	0.4
Clashscore	3.3	4.8	4.6
**Average B-factors (Å^2^)**			
all	69.6	61.4	98.2
macromolecules	65.3	55.7	94.1
RNA/ions	116.0	129.4	138.9
heme	-	-	116
solvent	54.1	35.9	41.2

^a^Values in parentheses refer to the highest resolution shell.

### SapA binds an endogenous ligand within its small and hydrophobic/neutral binding cavity

**Fig 3:**
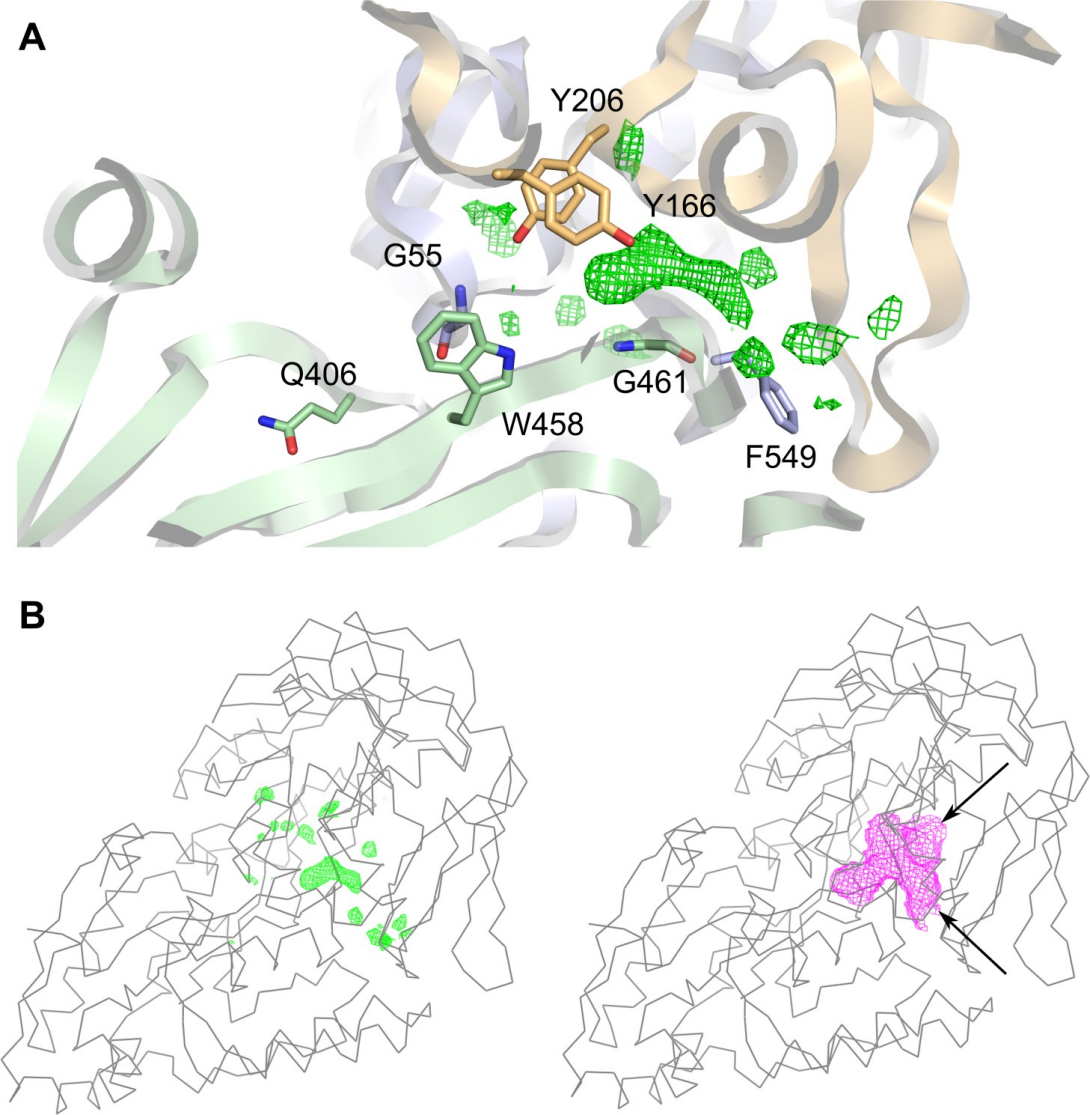
The SapA ligand-binding cavity. A, a detailed view of the SapA_closed_ ligand binding cavity. Aromatic residues lining the cavity are shown in stick representation together with the other residues discussed in the text. Unexplained *Fo-Fc* residual density is represented as a green mesh and is contoured at 3σ. B, left: The same *Fo-Fc* density highlighted in the context of the overall view of SapA_closed_ (cα trace). Right: The ligand-binding cavity of SapA_closed_ as calculated by the program *Voidoo* (magenta mesh). Arrows indicate cavity openings to the protein surface.

In SapA_closed_, the lobes enclose a cavity with two narrow openings to the protein surface. Difference electron density (*F*_o_—*F*_c_) became apparent within this cavity during the later stages of refinement (Fig 3A). This additional density was not observed in SapA_open_. Since the location of the SapA_closed_ cavity approximately coincides with the ligand binding cavities of other SBPs (Fig 2), we inferred that this density might represent an endogenous ligand likely derived from the cell lysate. The presence of an endogenous ligand rationalizes the observed closed conformation of SapA_closed_. The amino acids lining the ligand-binding cavity of SapA are predominantly hydrophobic or neutral (Table 2), the only exceptions are Glu 63, Lys 68 and Asp 71; however, these residues are located at the opening of the ligand binding cavity and may be classed as surface exposed.

**Table 2:** Residues in the putative ligand-binding cavity. Neutral or hydrophobic residues are underlined.

Domain	Residue Range	Cavity residues
domain 1b	(loop 55:68)	gly55, ser57, met58, asn59, val60, glu63, lys68
domain 1a	(loop 71:72)	asp71, ile72
domain 1a	(loop 166)	tyr166
domain 1a	(loop 200:206)	ser200, ala203, ser204, gln205, tyr206
domain 2	(loop 458:463)	trp458, leu459, ala460, gly461, asn462, leu463
domain 1b	(loop 547:552)	thr547, phe549, gly550, ser551, leu552

**Fig 4:**
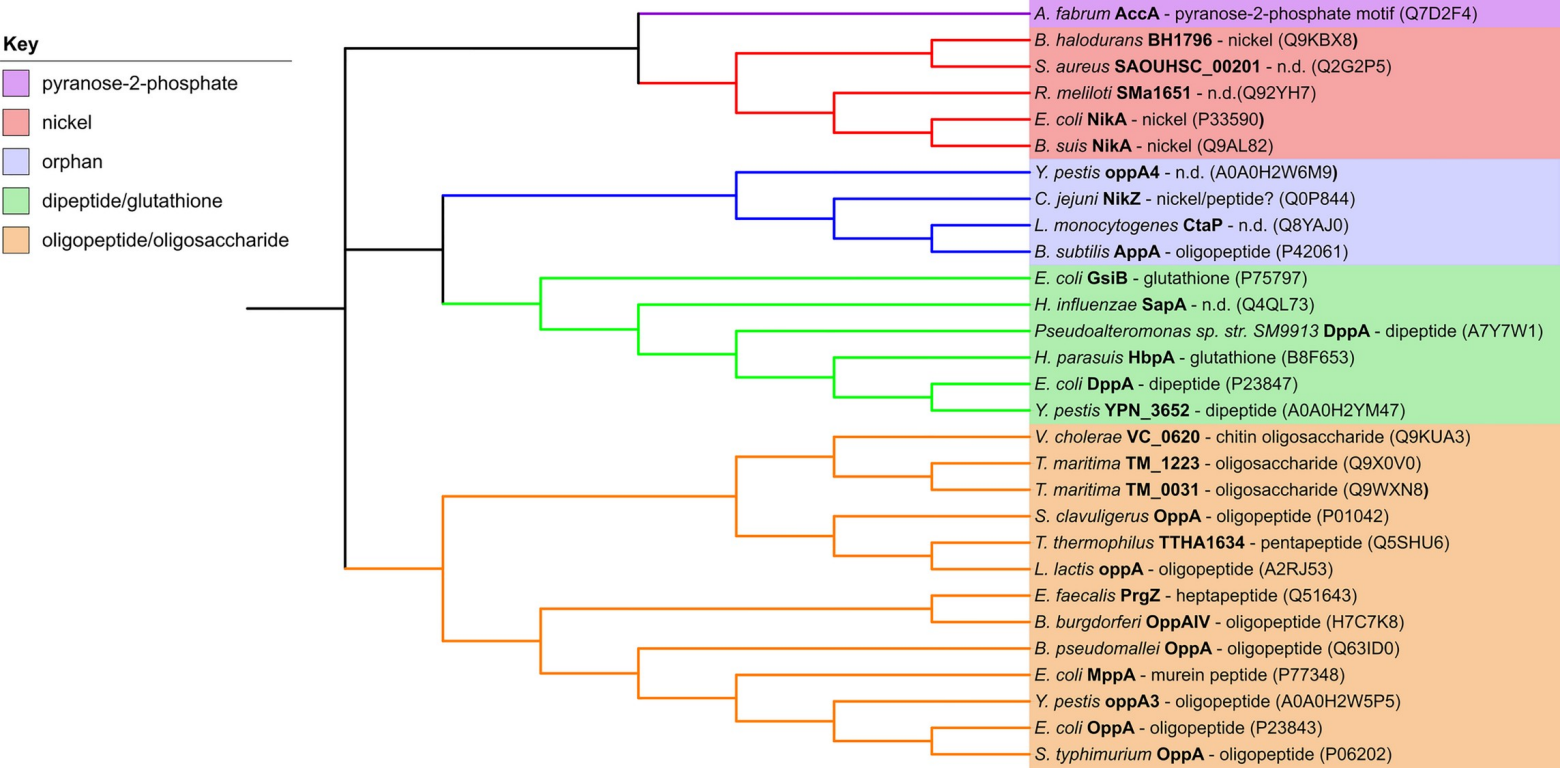
Cladogram of sequence relationships between structurally characterized SBP cluster C members. Clades organize by distinct functions that are colored according to the key.

Analysis of the ligand binding cavities across a range of peptide binding proteins structurally homologous to SapA carried out by [33] showed that cavity volumes correlate well with the size of peptides accepted by the binding site. For instance, *L*. *lactis* OppA (*Ll*OppA), which can bind peptides of up to 35 residues, has a very large cavity (4900 Å^3^), whereas dipeptide binding *Ec*DppA has a much smaller cavity (700 Å^3^). The SapA cavity volume is even smaller again at approximately 400 Å^3^, which implies that SapA can only bind a small ligand such as a single amino acid or dipeptide (Fig 3B). That SapA is likely to bind a ligand of this type is also predicted by phylogenetic analysis of structurally characterized SBP Cluster C members. Protein sequences of Cluster C members as described by [31], together with the sequences of NT*Hi*SapA and *Ps*DppA, were used to construct a cladogram of sequence relationships (Fig 4). SapA falls within a clade of proteins functionally described to bind either dipeptides or glutathione (a tripeptide).

### SapA binds heme with low affinity

**Fig 5:**
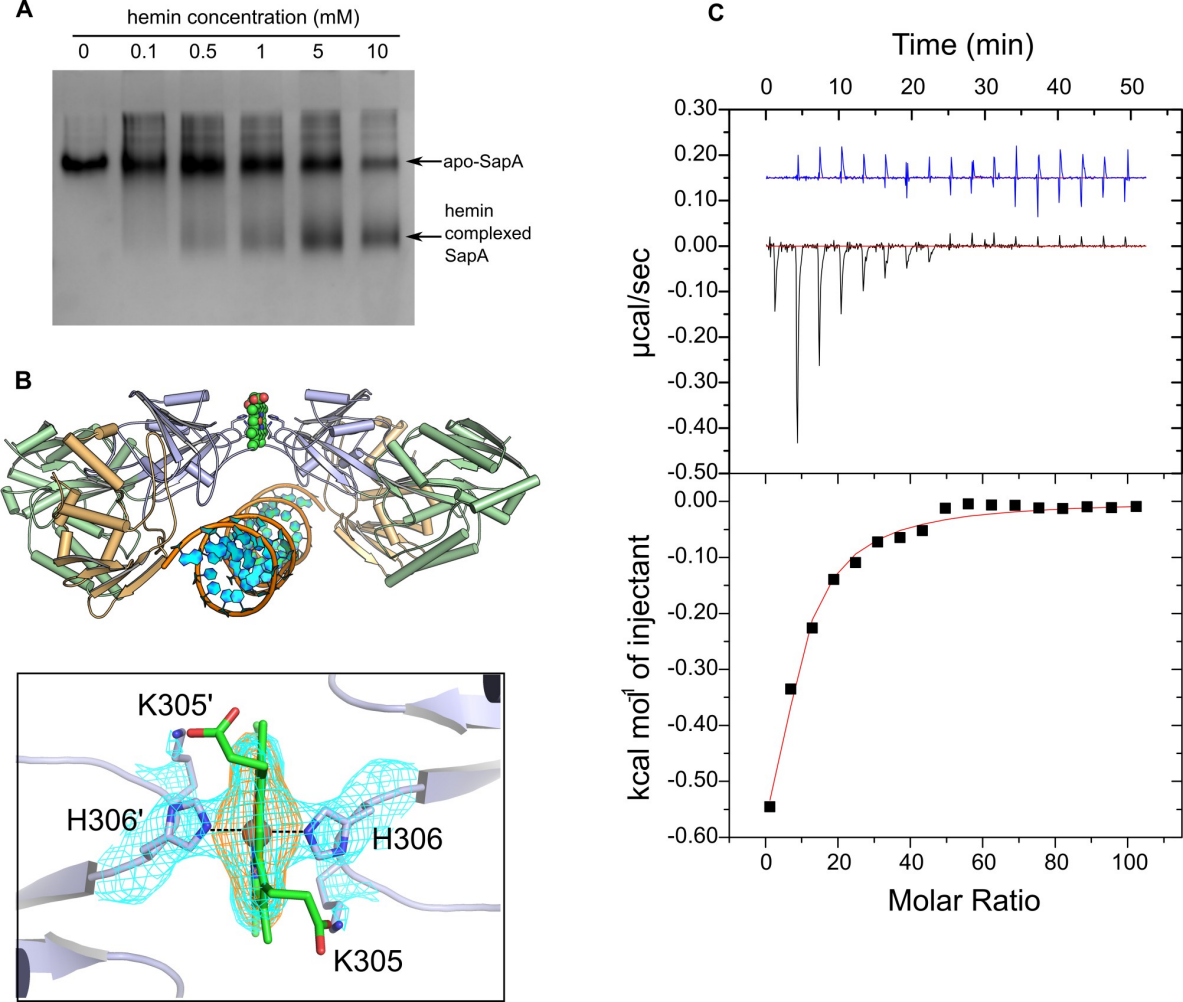
SapA interaction with heme. A, native gel assay for hemin interaction with SapA: Lanes 1–6 correspond to increasing concentrations of Hemin incubated with 5 μg of SapA. B, Soaking of hemin into SapA crystals. Top panel: a spherical representation of a heme molecule coordinated on the interface of two symmetry-related copies of the SapA_heme_ monomer. Bottom panel: the heme iron (orange sphere) is coordinated by His 306 (dashed line) from two symmetry mates. The meshes represent *2Fo-Fc* (cyan) and *Fo-Fc* (orange) maps contoured at 1 and 3.5σ respectively. C, isothermal titration calorimetry; Top panel: The experimental trace (black) obtained upon injection of hemin into SapA and into buffer control (blue). The baseline is indicated in red. Bottom panel: The integrated heats upon injection (black squares) and the data fit (red line) after subtraction of the control data.

Initial qualitative assessment of hemin/SapA interaction was carried out by Native PAGE assay (Fig 5A). The results are strongly suggestive of complex formation at high hemin concentration. Under the conditions of the assay, the interaction did not appear to be saturated before the limit of the hemin solubility was reached.

Hemin was also soaked into SapA crystals. From these a structure was obtained, SapA_heme_ (PDB = 7OFW). As with SapA_closed_, SapA_heme_ displayed difference density in the central ligand binding pocket. However, the pocket remains too small (approximately 400 Å^3^) to accommodate a substantial ligand such as heme, which occupies a volume closer to 800 Å^3^. Furthermore, additional difference density, corresponding to heme, was present on the protein surface. The bound heme forms a crystal contact between two symmetry-related SapA molecules, each coordinating the ligand via a surface exposed histidine (His 306 in both cases) (Fig 5B).

Isothermal titration calorimetry (ITC) was performed to quantitatively assess the binding affinity. A sigmoidal binding curve for this reaction was not observed for this reaction due to the binding affinity. Therefore, the binding stoichiometry of the reaction was fixed to 1, allowing a *K*_d_ of 282 ± 18.3 μM to be proposed (Fig 5C). Although the heme bound in the SapA_heme_ crystal structure is present at a crystal contact, providing a stoichiometry of 0.5, there are no further interactions between the protein molecules. Therefore, there is no reasonable basis to suggest that the 2 SapA:1 heme complex shown in Fig 5B is a physiologically relevant dimer. Control experiments indicated that the measured heats were not caused by dilution or hemin aggregation.

### SapA interacts with dsRNA

**Fig 6:**
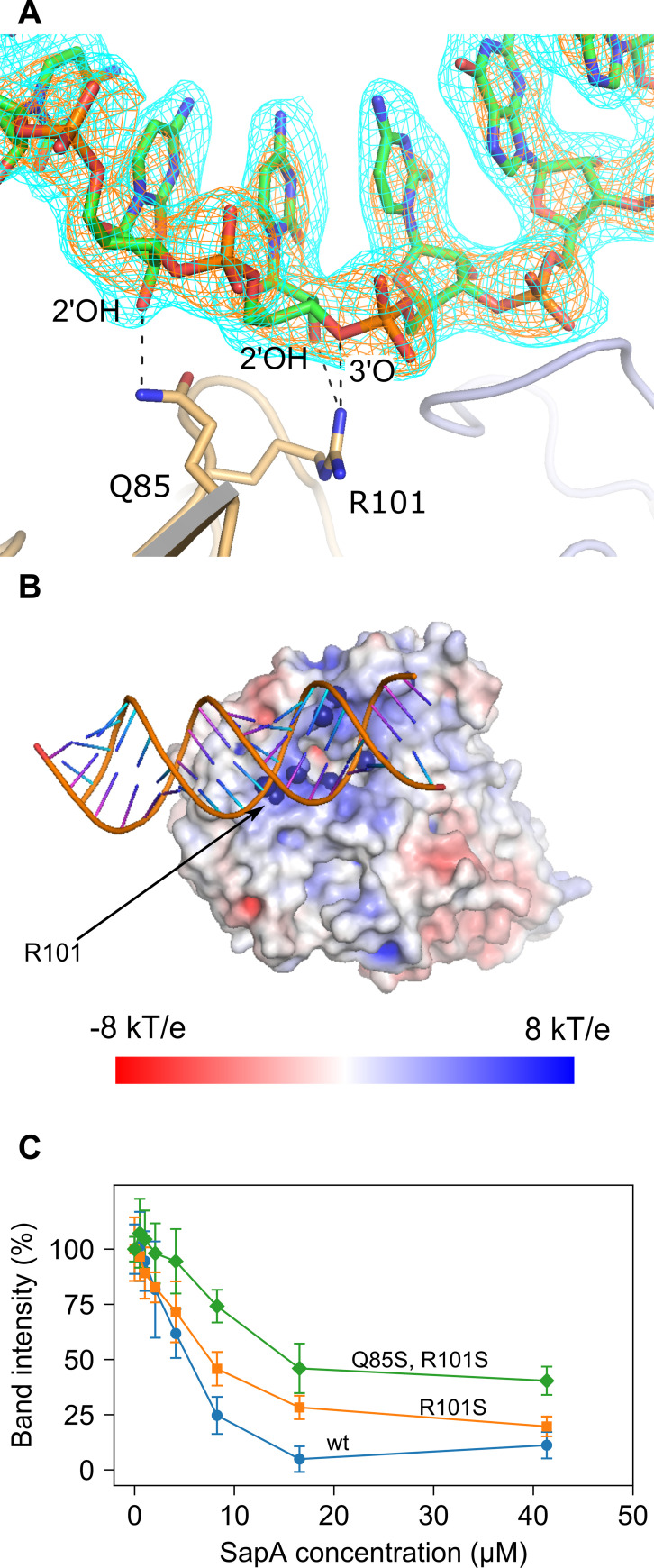
SapA interaction with RNA. A, residues in the Ia domain of SapA involved in hydrogen bonding (black dashed lines) to the RNA helix backbone. The meshes represent 2Fo-Fc (cyan) and Fo-Fc (orange) maps contoured at 1 and 2.5σ respectively. B, the surface of SapA coloured by electrostatic potential. NH1 and NH2 atoms of Arg 101 and NZ atoms of Lysines 73, 258, 259, 262, 542 and 545 are visualised as blue spheres. Electrostatic potentials (units of kT/e from −8 to 8) were calculated by APBS and visualized on the solvent-accessible surface by the program PyMOL (Version 1.5.0 Schrödinger, LLC). C, Agarose gel electrophoretic mobility shift assays: 0.5 μg of genomic E. coli DH5α RNA was incubated with increasing concentrations of wt SapA (blue line), single mutant SapA R101S (orange line) and double mutant SapA R101S, Q85S (green line). The intensities of the 23S and 16S rRNA bands were measured at each protein concentration. (See S2A Fig for typical gel data).

SapA co-crystallized with a strand of endogenously acquired RNA coordinated on the opposite face to the binding cleft. Identical RNA binding was observed at the same site in both SapA_closed_ and SapA_open_ and therefore did not appear to affect the overall conformation of the protein. The RNA crystallized as a double helix with parameters consistent for standard A-form dsRNA [34], with a radius of ~12 Å and pitch of 31 Å with 11 bp per turn and a rise per bp of 2.8 Å (31 Å/11 bp). The antisense strand is provided by a SapA symmetry mate. Furthermore, although definitive electron density is only present for a 19 bp stretch RNA, inspection of the symmetry neighbors indicates that the RNA strands extend across asymmetric units, forming a matrix within the crystal. The RNA backbone is coordinated to positively-charged chemical groups on the SapA surface. Specifically, the guanidino group of Arg 101 forms a hydrogen bond with the ribose 2’ hydroxyl and the 3’oxygen. Additionally, Gln 85 interacts with the O4 ring oxygen and 2’ hydroxyl of neighboring ribose residues (Fig 6A). APBS electrostatics analysis [35] of the SapA surface revealed that Arg 101 contributes to a wider positively charged patch formed by lysine residues–Lys 73, 258, 259, 262, 542 and 545 (Fig 6B). This area provides a counter-charge to the negatively charged RNA molecule. Interestingly, both Gln 85 and Arg 101 are located within the conserved SBP family 5 motif—(LIVM)**A**X_2_(WI)X_1_ or _2_(SN)(KE)**D**X_4_T(FY)X(LIV)**R**X_3_**K** - which in NT*Hi* SapA encompasses residues 84–106 of domain Ib. This motif represents a signature sequence within a system of classification based on sequence similarity where most SBPs can be grouped into 8 families or clusters. Family 5 nominally includes binding proteins for peptides and nickel [36] such as *E*. *coli* oligopeptide, dipeptide, murein, and nickel binding proteins OppA, DppA, MppA, and NikA. Besides SapA in *H*. *influenzae* it includes the periplasmic lipoprotein HbpA.

The ribonucleotides were modeled as C-G pairs because at 2.6 Å resolution it was not possible to assign the exact sequence of individual bases. The density accounting for the RNA showed signs of possible density averaging between the two stands suggesting that heterogenous RNAs bind this site.

To determine the specificity of Arg 101 and Gln 85 in binding RNA, single (Arg101Ser) and double (Gln85Ser and Arg101Ser) serine mutants were generated and tested by agarose gel electrophoretic shift assay (EMSA). Here the wild-type and mutant proteins were assayed for interaction with DH5α *E*. *coli* ribosomal RNA, as measured by the decrease in intensity of 23S and 16S rRNA bands at increasing protein concentrations. The double mutant showed the greatest reduction in binding relative to the wild-type, whilst the single mutant showed an intermediate effect (Fig 6C, S2A Fig). Dissociation constants (*K*_d_) could be estimated from these experiments as 4.4 μM for wild-type, 7.7 μM for single and 23.0 μM for double mutant SapAs.

The mutants were also tested for DH5α *E*. *coli* genomic DNA binding. The interaction was only observed at the highest protein concentrations tested (41 μM) with no significant difference between the wild-type and mutant proteins (S2B Fig). Overall, these results indicate that Arg 101 and Gln85 are responsible for the specific binding to RNA rather than contributing to a general electrostatic effect by providing a counter charge to the anionic nucleic acids.

## Discussion

SapA was successfully crystallized in an open (no ligand) and closed conformation with an endogenous ligand in the binding cavity. The cavity volume of SapA_closed_ and the unexplained electron density within it can potentially accommodate a small ligand such as a short peptide or even an extended polypeptide chain that could protrude out of the narrow openings of the SapA ligand-binding cavity. However, there is not enough cavity volume to accommodate AMPs in their folded state. Additionally unfolding of BDs would require disruption of disulfide bridges. SapA_closed_ has a cavity volume of 400 Å^3^ whereas LL-37 and hBDs have volumes ranging from 3000 Å^3^ to 8500 Å^3^. As discussed previously, the SapA binding cavity is formed predominantly by hydrophobic or neutral residues, providing no obvious countercharges for the reported cationic AMP ligands of SapA (Fig 3A, Table 2). We found no evidence for hBD1, hBD2, hBD3 and LL-37 binding to highly purified SapA protein by crystallographic or biophysical methods (ITC, thermofluor; S1 File Supporting methods).

We suggest the most likely ligand occupying the binding cavity of SapA_closed_ is a hydrophobic di/tripeptide based on overlap predictions from cavity size volume, the hydrophobic nature of the cavity and phylogenetic analysis. We attempted many experiments to confirm binding of dipeptides, however were unable to do so, probably due to some of our sample already being in a ligand occupied state preventing further interaction. Our efforts to devise a denature/refold protocol followed by ligand screening were unsuccessful and additionally complicated by low SapA expression levels.

It should be noted that while NT*Hi*SapA organizes into a functional clade with di/tripeptide binding SPBs, there is poor conservation of ligand-binding residues with its homologs *Ps*DppA (33% identity), *Ec*DppA (30% identity) and *Hp*HbpA (31% identity) (S3 Fig). In the DppA SBP family, strongly conserved binding site aspartate and arginine residues (Asp 436, Arg 383 in *Ec*DppA, Asp 432, Arg 379 in *Hp*HbpA) confer specificity for peptides by coordinating the dipeptide terminal carboxyl and amino moiety respectively. However, in SapA, neither are conserved, with the aspartate replaced by Gly 461 and the arginine replaced by Gln 406, with the Gln 406 side chain oriented out of the binding site (Fig 3A). In SapA, the tip of the loop centered on Gly 55 replaces the approximate volume occupied by the ligands in DppA and HbpA, effectively bisecting the binding cleft (Fig 2). As a result, the endogenous SapA ligand lies shifted along the cleft relative to the DppA and HbpA ligands, in a cavity that is much more reduced in volume. The possibility that SapA binds something other than a short peptide therefore cannot be ruled out. Cluster C SBPs, albeit more phylogenetically distant ones, also bind nickel, longer oligopeptides, oligosaccharides and pyranose-2-phosphate containing compounds (Fig 4).

We can confirm that NT*Hi*SapA directly binds heme in agreement with other studies [17, 19]. In addition to native gel and ITC evidence we provide structural evidence for heme binding on the surface of the SapA molecule. This surface location is far from the ligand binding cavity and out of the context of any defined pocket and may therefore cast some doubt on the physiological relevance of this interaction. Nevertheless, at the very minimum, we can conclude that a surface residue His 306 has the capacity to coordinate heme to the SapA molecule. It is the first molecular-detail experimental structural evidence for heme binding to SapA or indeed any Cluster C SBP. SapA homologs *Ec*DppA, *Ec*MppA and NT*Hi*HbpA have also been reported to bind heme but without direct structural evidence. Consequently, heme site predictions have relied on computational docking [17–19, 37]. Our observation that the heme site is physically separate from the ligand binding cavity in NT*Hi*SapA correlates well with the results of a SPR competition assay carried out with NT*Hi*OppA [19]. Here the authors showed that heme does not directly compete with peptide in the substrate-binding pocket. Their second prediction that, while separate, the two sites are in close proximity is not supported by our data. This discrepancy may be due to the limitations of computational docking and in the case of SapA-heme modelling [18] exacerbated by the previous unavailability of a crystal structure.

Questions remain surrounding the physiological relevance of RNA binding to SapA. The *K*_d_ of the RNA interaction is approximately 4.4 μM, 60 fold tighter than the interaction measured here with heme. This *K*_d_ falls within the range of measured binding affinities reported for 73 structurally characterized RNA-protein complexes [38]. Additionally, the RNA interaction is within a conserved sequence motif, which can be indicative of a functional role [39]. While there are no reports of RNA specifically localizing to the periplasm, RNA may conceivably exist in this compartment during processes such as viral infection and during the recently described bacterial secretion of small non-coding RNAs [40]. Under these circumstances, periplasmic SapA could encounter RNA. We can only speculate on what would be the function of such an interaction. Perhaps RNA binding motifs present on bacterial periplasmic proteins can act as "sticky patches" to prevent or slow down the entry of viral RNA as a mechanism of bacterial resistance to viral infection.

RNA contamination of the SapA protein may explain previous observations regarding AMP binding. Cationic AMPs interact with a variety of anionic macromolecules including nucleic acids [41–45]. Therefore, interactions previously observed between SapA and AMPs [14] may in fact be via interactions with contaminating RNA bound to the SapA sample, with the RNA acting as the bridging molecule. This model would explain the results of a competition assay [17] where the ability of different AMPs to displace heme from SapA was correlated to their overall positive charge. Such observations are consistent with charge-charge interactions between molecules rather than a sequence-specific interaction characteristic of peptide/protein binding. Such effects were not apparent in our experiments as we ensured that our SapA samples avoided RNA contamination. The A_260_/A_280_ ratio of purified SapA samples was monitored; and a measured value of ~0.6 was taken to be consistent with a nucleic acid free sample. In this work, we utilized a number of techniques using highly purified components to characterize SapA and the molecular interactions with its proposed ligands. The weight of our evidence favors the involvement of SapA, and its cognate transporter, in the transport of dipeptides or tripeptides, even though further research will be required to confirm this on a functional level. Research should take two distinct directions: one, to confirm the true substrate of the transporter and two, to elucidate the mechanisms of AMP resistance. That AMP sensitivity may not be mediated through direct molecular interactions with Sap needs to be considered, given that *sapA* sensitivity to AMPs is not conserved across species nor is it confined to a particular set of AMPs. For instance, the *H*. *ducreyi sapA* mutant was more sensitive to LL-37 but not human defensins [22] while resistance to LL-37 in *E*. *coli* was unaffected by Δ*sapBCDF* deletion [46]. In closing, the data presented here provide the basis for the community to cast a wider net and consider other mechanisms and pathways that could explain the observed heme requirement and AMP sensitivity of NT*Hi sap* genetic mutants in cell assays and *in vivo*.

## Materials and methods

### NT*Hi* SapA protein expression and purification

Purified NT*Hi* 86-028NP genomic DNA (gift of Kevin Mason, Nationwide Children’s Hospital, Columbus, Ohio, USA) served as a template for PCR amplification of the SapA protein-coding region. The In-Fusion® cloning method was used to insert the PCR product into a pOPINF plasmid vector [47]. The construct was designed to exclude the predicted signal peptide region and encompassed residues 33 to 560 with a S3C cleavage site. The recombinant plasmid was transformed into *E*. *coli* Rosetta^TM^ (DE3) expression strain (Novagen). A single colony was used to inoculate 100 ml of Power *Prime* Broth™ media (Molecular Dimensions) supplemented with 50 μg/ml carbenicillin, 35 μg/ml chloramphenicol and grown overnight at 37°C, 230 rpm. 10 ml of the overnight culture was then used to inoculate 1 liter of Overnight Express™ Instant TB autoinduction media (Novagen). The cultures were initially grown at 37°C for 5–6 h, followed by prolonged growth at lowered temperatures (230 rpm, 20 h, 25°C). The cells were harvested by centrifugation and stored at -80°C. Approximately 50 g of cells were resuspended in lysis buffer (50 mM Hepes pH 8, 500 mM NaCl, 30 mM imidazole pH 8, 0.2% Tween-20, 5% (w/v) glycerol) supplemented with DNase I (10–20 μg/ml) and a cOmplete^™^ EDTA-free Protease Inhibitor Cocktail tablet (Roche), and lysed using a Constant Systems Ltd. cell disrupter (3 passes, 30 kpsi, 4°C). The crude extract was centrifuged (50000 g, 1hr, 4°C) and filtered with a 0.22 μm filter to remove insoluble components.

The soluble lysate was loaded onto a 1 ml nickel sepharose HisTrap FF column (GE Healthcare) equilibrated in wash buffer (50 mM Hepes pH 8, 500 mM NaCl, 30 mM imidazole pH 8, 5% (w/v) glycerol). The bound protein was washed using 20 column volumes of wash buffer and eluted using an Imidazole step gradient using the wash buffer containing 500 mM Imidazole.

The major peak from Ni-affinity purification was collected and applied to a HiLoad 16/600 Superdex 200 pg size-exclusion column (GE Healthcare) equilibrated in 20 mM Hepes pH 8, 500 mM NaCl and 5% (w/v) glycerol. Post size-exclusion, the protein was subjected to (His)_6_ tag cleavage by incubation with 50% (w/w) Human Rhinovirus B 3C protease (2 h at 20°C then 14 h at 4°C), followed by reverse His-tag purification.

Purity at all stages was monitored by SDS-PAGE. High purity samples were pooled and concentrated to 10 mg/ml for crystallization using a 10 kDa MWCO Amicon-Ultra centrifugal filter unit (Merck). In parallel, the buffer was exchanged on the unit (final buffer: 20 mM Hepes pH 8, 500 mM NaCl). The molecular weight of the purified protein was confirmed by intact mass spectrometry (Predicted Mass / Experimental Mass = 60460.64 / 60458.29 Da).

### Crystallisation, data collection and structure determination

Diffraction quality crystals (SapA_closed_) were obtained from 200 nl sitting drops where the protein was mixed 1:1 with 22% (w/v) PEG 3350, 0.25 M NaBr, 0.1 M Bis-Tris Propane pH 7.5.

In an attempt to obtain RNA-free crystals the above procedure was modified by adding 0.5 ml of RNase Cocktail™ Enzyme Mix (Ambion) to the clarified and filtered cell lysate. This was incubated on ice for 1 hour prior to Nickel sepharose purification. A second crystal form, SapA_mixed_, was subsequently obtained from this protein preparation, in a crystallization condition containing 10% (w/v) PEG 8000, 0.1 M imidazole pH 8.0, 0.2 M Ca(OAc)_2._ Heme derivative SapA_heme_ crystals were obtained by soaking closed-form crystals for 48 h in a mother liquor solution containing Hemin added to a theoretical concentration of 25 mM. Hemin was added from a freshly prepared unfiltered suspension generated by the addition of hemin to 0.5 M NaOH and subsequent neutralization to pH 8. A theoretical concentration of 25 mM hemin was also maintained in the cryoprotectant solution.

The crystals were then rapidly transferred from the sitting droplet into a cryoprotectant solution, the composition of which was based on the original crystal growth condition, and either 20% (v/v) glycerol or ethylene glycol. The crystals were then immediately plunged into liquid nitrogen. X-ray diffraction data were collected at 100 K at beamline I04 and I04-1, Diamond Light Source (Didcot, UK). Data were processed using the xia2 automated reduction pipeline [48] which makes use of Mosflm [49], Pointless [50], CCP4 [51] and XDS [52].

The structure of SapA was solved by molecular replacement by the programs BALBES [53] and Phaser [54] in the CCP4 suite of programs. The molecular replacement pipeline BALBES identified the crystallographic structure 3M8U as the best search model. Initial model autobuilding and refinement was carried out in PHENIX [55]. Further rounds of manual building and refinement were carried out using the programs *Coot* [56], Refmac5 [57], BUSTER [58] and the PDB-REDO server [59]. The quality of the final model was assessed with the PDB Validation Services and MolProbity [60]. Domain movements were analyzed using ProSMART [61] by aligning the structure of SapA_closed_ with chain B of SapA_mixed_. Structural alignment with SapA homologs was carried out using the SSM superpose tool within the program *Coot*. Sap cavity volume calculations were carried out using the program Voidoo [62] using the same parameters as described by [33]. Identification of residues lining the cavity was carried out with the same program.

### Phylogenetic analysis

The phylogenetic analysis used structurally characterised SBP cluster C members as described in Scheepers *et al* with NT*Hi*SapA and *Ps*DppA added to the set. UniProt derived sequences for this set were aligned with Clustal Omega [63]. Phylogenetic neighbour joining tree data we obtained from within Clustal Omega and the corresponding cladogram was visualised using iTOL [64] software.

### Native PAGE gel shift assays

Samples for native PAGE were prepared by mixing 5 μg of purified SapA protein with 0, 0.1, 0.5, 1, 5, 10 mM hemin from a freshly prepared concentrated hemin stock. The volumes were adjusted to a final volume of 20 μl by the addition of 20 mM Hepes pH 8, 300 mM NaCl buffer. The samples were then incubated for 45 min at room temperature, prior to separation by native PAGE (10% gel, running buffer pH 8.8) as previously described [65, 66].

### Isothermal titration calorimetry

Isothermal titration calorimetric measurements were carried out using a MicroCal iTC200 microcalorimeter (Malvern Instruments UK) at 25°C. 200 μL of SapA (20 μM) in was placed in the cell and 40 μL of hemin (10 mM) in the syringe. The buffer used in ITC was 20 mM Hepes pH 8, 300 mM NaCl. The concentration of SapA and hemin solutions were determined using spectrophotometry at 280 and 385 nm, and the extinction coefficients used were ε_280_ = 77490 M^-1^cm^-1^ and ε_385_ = 58440 M^-1^cm^-1^, respectively. Sixteen 2.4 μL injections were performed at an injection speed of 0.5 μL/sec, with a pre-injection of 0.5 μL, a three-minute interval between injections and a stirrer speed of 750 rpm. To establish the heat of dilution, a control experiment was performed where hemin (10 mM) was injected into the ITC buffer using identical experimental conditions. This was then subtracted from the main experiment. Data were analyzed using MicroCal Origin software (version 7) fitting to a single site binding model. The binding enthalpy (Δ*H*) and association constant (*Ka*), were permitted to float during the least-squares minimization process and taken as the best-fit values. The binding stoichiometry (*n*) was fixed to one.

### RNA and DNA electrophoretic mobility shift assay (EMSA)

SapA R101S and SapA Q85S R101S mutants were generated for the purpose of testing in EMSAs. The double-stranded DNA encoding the mutant proteins was purchased as gBlocks® Gene Fragments (Integrated DNA Technologies) and cloned into pOPINF plasmids by the In-Fusion® method. Purification was carried out as for the wild-type protein.

0.5 μg of genomic total RNA from *E*. *coli* DH5α (Ambion) was incubated with increasing amounts of wild-type SapA, SapA R101S and SapA Q85S R101S mutants in a total volume of 20 μl of TBE at room temperature for 1 hour. Following incubation, the samples were loaded on a 1% (w/v) agarose/TBE gel containing SYBR™ Safe Gel Stain (Invitrogen) at 1:10000 dilution. The samples were then separated at 60 V for 1 hour in 1X TBE buffer. The gels were then imaged and bands were quantified using the ImageQuant TL 1D v8.1 software. The background subtracted intensities for rRNA 16S and 23S bands were summated, normalized against the average intensity at zero concentration and plotted. The experiments were repeated 4 or 5 times. The *K*_d_ of an interaction was estimated by least squares linear regression of the normalized reduction in rRNA band intensity using Origin (version 7) fitting to the equation:

$$PR=\frac{Kd+[P]+[R]-\sqrt{{\left(Kd+[P]+[R]\right)}^{2}-4[P][R]}}{2}$$

where PR is the normalized reduction in rRNA band intensity, and [P] and [R] are the concentration of the SapA protein and the rRNA, respectively.

Total genomic DNA was isolated from Subcloning Efficiency™ DH5α™ Competent Cells (Invitrogen) using the GenElute™ Bacterial Genomic DNA Kit (Sigma) according to the manufacturer’s instructions. The genomic DNA was then concentrated by vacuum concentration (to approximately 200 ng/μl) and dialyzed into water using a 3500 MWCO Slide-A-Lyzer MINI Dialysis Device (Pierce). The assays were carried out as above with the following modifications: Protein samples were diluted in TAE and incubated with 0.5 μg of gDNA for 1 hour. Electrophoresis was carried out at 75 V for 30 minutes on a 0.8% (w/v) agarose/TAE gel.

## Supporting information

S1 File Supporting methods: Expression and purification of human beta defensins and isothermal titration calorimetry of AMPs. (DOCX)

S1 Fig Surface representations of SapA structures. A, SapA_closed_. B, SapA_mixed_. C, SapA_heme_. RNA and heme are shown in sphere representation and protein chains A and B are colored gray and yellow respectively.
(DOCX)

S2 Fig Typical EMSA results for the interaction of SapA with nucleic acids on agarose gels. A, DH5α *E*. *coli* gRNA incubated with increasing concentrations of wt SapA and mutants of SapA. Arrows on the left mark the position of rRNA bands and asterisks indicate starting well position B, DH5α *E*. *coli* gDNA incubated with increasing concentrations of BSA, wt SapA and SapA mutants.
(DOCX)

S3 Fig Alignment of amino acid sequences of NT*Hi* SapA (UNIPROT = Q4QL73) with its closest structural homologs *Hp*HbpA (UNIPROT = B8F653), *Ec*DppA (UNIPROT = P23847) and *Pseudoaltermonas* sp. SM9913 DppA (UNIPROT = A7Y7W1). Non-conserved NTHi SapA residues discussed in the main text are highlighted in solid green. α-helices are displayed as squiggles respectively. β-strands are rendered as arrows, strict β-turns as TT letters. Figure generated with *ESPript 3*.*0* [67].
(DOCX)

S1 Raw images (PDF)

## Abbreviations

NT*Hi*: nontypeable *Haemophilus influenzae*
COPD: chronic obstructive pulmonary disease
OM: otitis media
Sap: sensitivity to antimicrobial peptides
AMP: antimicrobial peptide
SBP: substrate-binding protein
hBD: human Beta-Defensin
cBD: chinchilla Beta-Defensin
ITC: isothermal titration calorimetry
EMSA: electrophoretic mobility shift assay
Ll: *L*. *lactis*
Ec: *E*. *coli*
Hp: *H*. *parasuis*
