# Supplementary material for: The structure of nontypeable Haemophilus influenzae SapA in a closed conformation reveals a constricted ligand-binding cavity and a novel RNA binding motif
Source: PLoS One. 2021 Oct 15;16(10):e0256070. doi: 10.1371/journal.pone.0256070 (PMC8519434; doi:10.1371/journal.pone.0256070)
Supplement: S1 File — (DOCX) [file pone.0256070.s001.docx]

**SUPPORTING INFORMATION**

**S1 Supporting Methods**

**Expression and Purification of Human Beta Defensins**

Human defensins were cloned into pET-50b(+) vectors (Novagen) as 6-histidine-tagged, HRV 3C protease cleavable, thioredoxin fusions (6His-TRX-3C-hBD). 1 μl of the vector was transformed into Lemo21(DE3) competent *E. coli* and plated on LB-agar plates containing 50 μg/ml Kanamycin and 35 μg/ml Chloramphenicol. The plates were incubated overnight at 37 °C and the next day a single colony was used to inoculate an LB starter culture containing 50 μg/ml Kanamycin and 35 μg/ml Chloramphenicol. 10 ml of the overnight culture was then used to inoculate 1 litre of Overnight Express™ Instant TB autoinduction media (Novagen). The cultures were initially grown at 37 °C, followed by prolonged growth at lowered temperatures (230 rpm, 20 h, 25 °C). The cells were harvested by centrifugation and stored at -80 °C. Cells were resuspended in lysis buffer (20 mM Hepes pH 7.5, 500 mM NaCl, 30 mM Imidazole, 0.2 % (v/v) Tween-20) supplemented with DNase I (10-20 µg/ml) and a cOmplete^™^ EDTA-free Protease Inhibitor Cocktail tablet (Roche), and lysed using a Constant Systems Ltd. cell disrupter (3 passes, 30 kpsi, 4 °C). The lysate was centrifuged (50000 g, 1 hr, 4 °C) and then filtered with a 0.22 µm filter. The lysate was applied to a 5 ml nickel sepharose HisTrap FF column (GE Healthcare) and the column was washed with 20 volumes of 500 mM NaCl, 20 mM Hepes pH 7.5, 30 mM Imidazole. Step elution of the protein was then carried out with the same buffer containing 500 mM Imidazole. The protein in the elution peak was collected and mixed with Human Rhinovirus B 3C protease at 50:1 molar ratio. The mixture was placed in 2000 Da MWCO Slide-A-Lyzer™ dialysis cassette and dialysed overnight against 500 mM NaCl, 20 mM Hepes pH 7.5 at 4 °C. The next day the sample was passed three times over a 5 ml HisTrap FF column before carrying out a reverse His-tag purification. High-purity samples from this step were concentrated on a 3500 Da MWCO Amicon-Ultra centrifugal filter unit (Merck). In parallel buffer exchange into 20 mM Hepes pH 7.5 was carried out on the concentrator to reduce the NaCl concentration of the sample to less than 20 mM. The desalted sample was applied to a cation-exchange 1 ml HiTrap CM Sepharose FF column (GE Healthcare) equilibrated in 20 mM Hepes pH 7.5. The protein was eluted with a 20 ml linear gradient of 0-0.5 M NaCl in the same buffer. High purity fractions were pooled, concentrated to 0.2-2 mg/ml and in parallel buffer exchanged into 100 mM NaCl, 20 mM Hepes pH 8 before carrying out ITC experiments. Molecular weights of the defensins and disulphide formation were confirmed by mass spectrometry.

**Isothermal Titration Calorimetry of AMPs**

The antimicrobial peptides LL-37 (AnaSpec), hBD1, hBD2, and hBD3 were tested. Isothermal titration calorimetric measurements were carried out using a MicroCal iTC200 microcalorimeter (Malvern Instruments UK) at 25 °C. 200 µL of SapA (20-100 µM) was placed in the cell and 40 μL of dipeptide/antimicrobial peptide (200-2000 µM) in the syringe. The concentration of SapA and beta-defensin solutions were determined using spectrophotometry at 280 nm. The concentration of dipeptide and LL-37 solutions were determined on the basis of the weight of lyophilised material assuming a 20% (w/w) correction for H_2_O content. Sixteen 2.4 µL injections were performed at an injection speed of 0.5 μL/sec, with a pre-injection of 0.5 µL, a three-minute interval between injections and a stirrer speed of 750 rpm. Data were analysed using NITPIC and SEDPHAT, fitting to the A + B ↔ AB hetero-association model.
