# Supplementary material for: The structure of nontypeable Haemophilus influenzae SapA in a closed conformation reveals a constricted ligand-binding cavity and a novel RNA binding motif
Source: PLoS One. 2021 Oct 15;16(10):e0256070. doi: 10.1371/journal.pone.0256070 (PMC8519434; doi:10.1371/journal.pone.0256070)
Supplement: S1 Fig — A, SapAclosed. B, SapAmixed. C, SapAheme. RNA and heme are shown in sphere representation and protein chains A and B are colored gray and yellow respectively. (DOCX) [file pone.0256070.s002.docx]

**
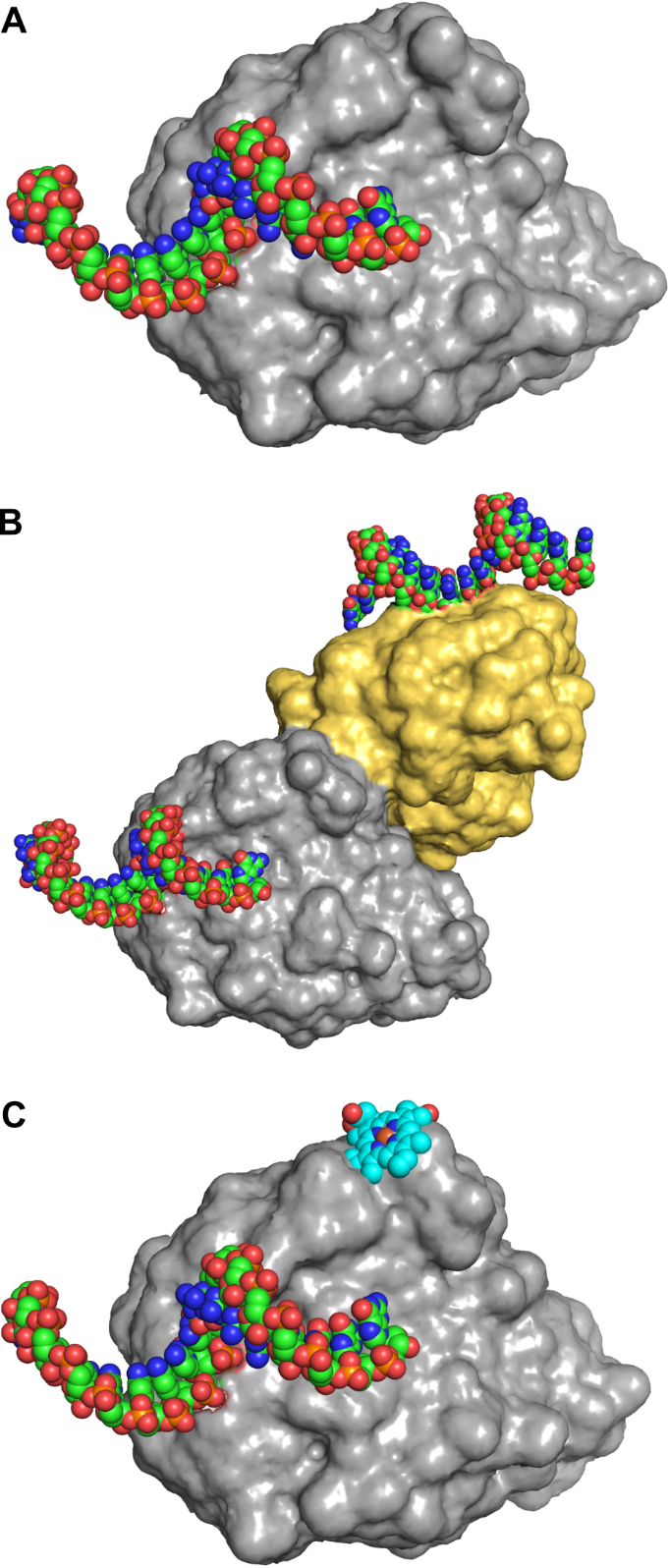
**

**S1 Fig. Surface representations of SapA structures.** A, SapA_closed_. B, SapA_mixed_. C, SapA_heme_. RNA and heme are shown in sphere representation and protein chains A and B are colored gray and yellow respectively.
