# Supplementary material for: The structure of nontypeable Haemophilus influenzae SapA in a closed conformation reveals a constricted ligand-binding cavity and a novel RNA binding motif
Source: PLoS One. 2021 Oct 15;16(10):e0256070. doi: 10.1371/journal.pone.0256070 (PMC8519434; doi:10.1371/journal.pone.0256070)
Supplement: S2 Fig — A, DH5α E. coli gRNA incubated with increasing concentrations of wt SapA and mutants of SapA. Arrows on the left mark the position of rRNA bands and asterisks indicate starting well position B, DH5α E. coli gDNA incubated with increasing concentrations of BSA, wt SapA and SapA mutants. (DOCX) [file pone.0256070.s003.docx]

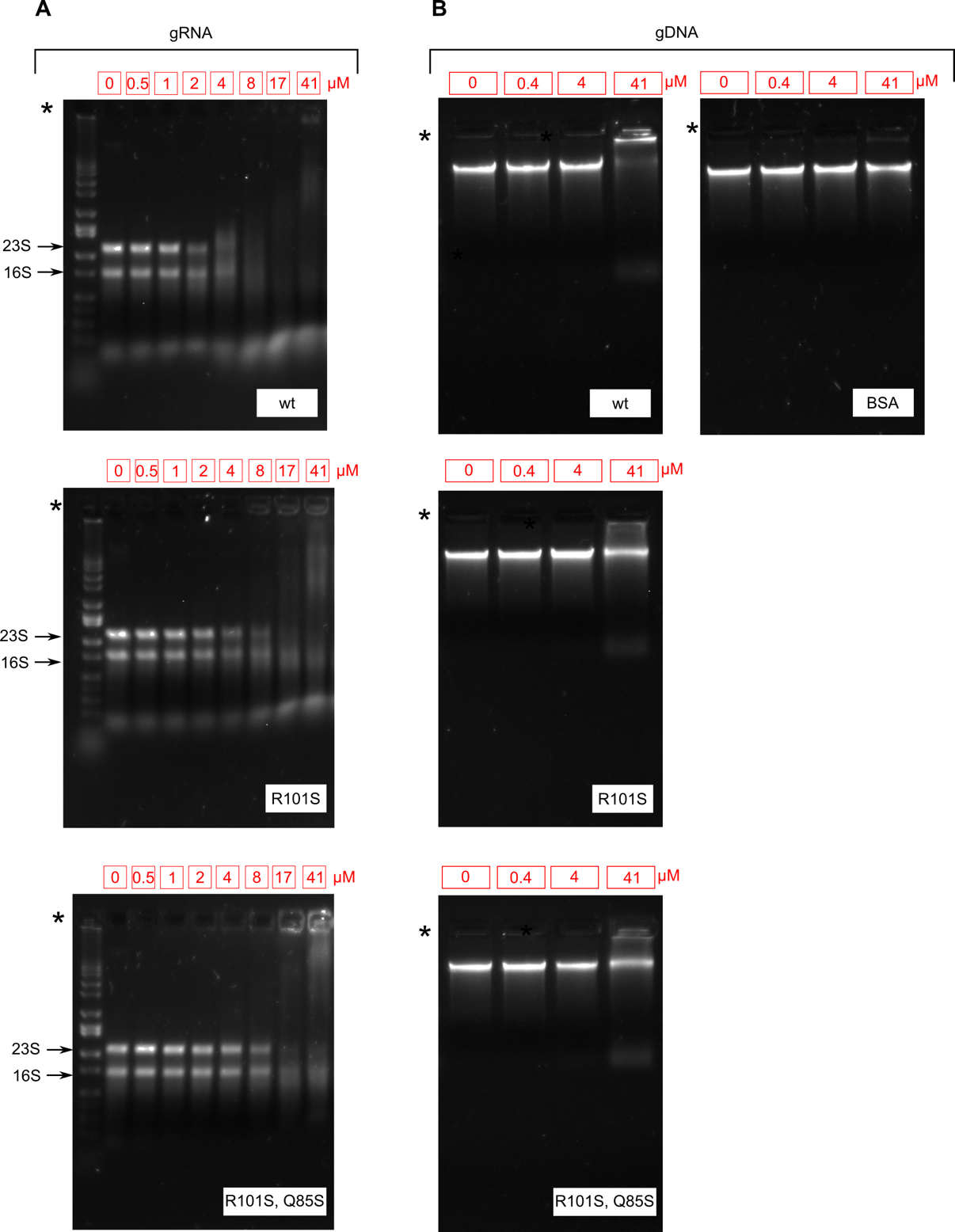


**S2 Fig. Typical EMSA results for the interaction of SapA with nucleic acids on agarose gels.** A, DH5α *E. coli* gRNA incubated with increasing concentrations of wt SapA and mutants of SapA. Arrows on the left mark the position of rRNA bands and asterisks indicate starting well position B, DH5α *E. coli* gDNA incubated with increasing concentrations of BSA, wt SapA and SapA mutants.
