# Supplementary material for: The structure of nontypeable Haemophilus influenzae SapA in a closed conformation reveals a constricted ligand-binding cavity and a novel RNA binding motif
Source: PLoS One. 2021 Oct 15;16(10):e0256070. doi: 10.1371/journal.pone.0256070 (PMC8519434; doi:10.1371/journal.pone.0256070)
Supplement: S3 Fig — Non-conserved NTHi SapA residues discussed in the main text are highlighted in solid green. α-helices are displayed as squiggles respectively. β-strands are rendered as arrows, strict β-turns as TT letters. Figure generated with ESPript 3.0 [67]. (DOCX) [file pone.0256070.s004.docx]

**S3 Fig. Alignment of amino acid sequences of NT*Hi* SapA (UNIPROT=Q4QL73) with its closest structural homologs** *Hp*HbpA (UNIPROT=B8F653), *Ec*DppA (UNIPROT=P23847) and *Pseudoaltermonas* sp. SM9913 DppA (UNIPROT=A7Y7W1). Non-conserved NTHi SapA residues discussed in the main text are highlighted in solid green. α-helices are displayed as squiggles respectively. β-strands are rendered as arrows, strict β-turns as TT letters. Figure generated with *ESPript 3.0*[67]*.*
