# Supplementary figures and images for: The structure of nontypeable Haemophilus influenzae SapA in a closed conformation reveals a constricted ligand-binding cavity and a novel RNA binding motif
Source: PLoS One. 2021 Oct 15;16(10):e0256070. doi: 10.1371/journal.pone.0256070 (PMC8519434; doi:10.1371/journal.pone.0256070)

Figure 1

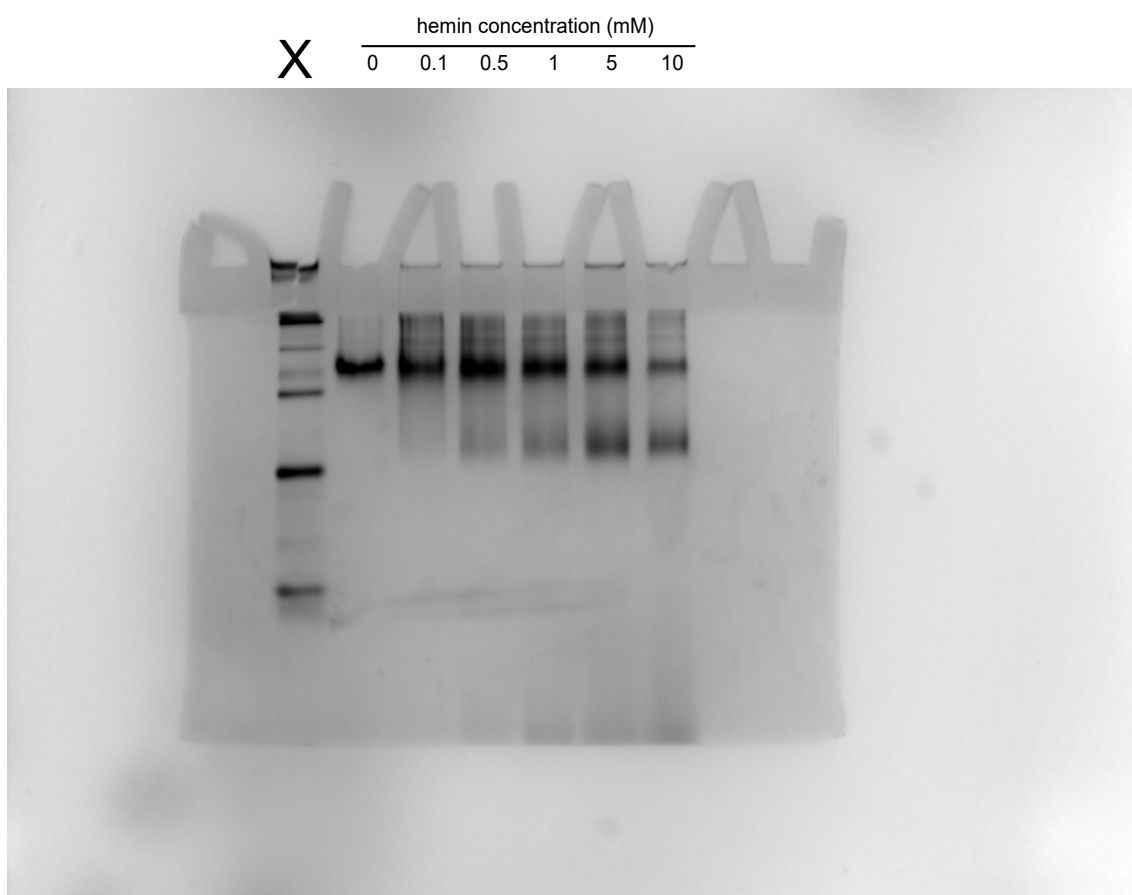

Figure S2 A

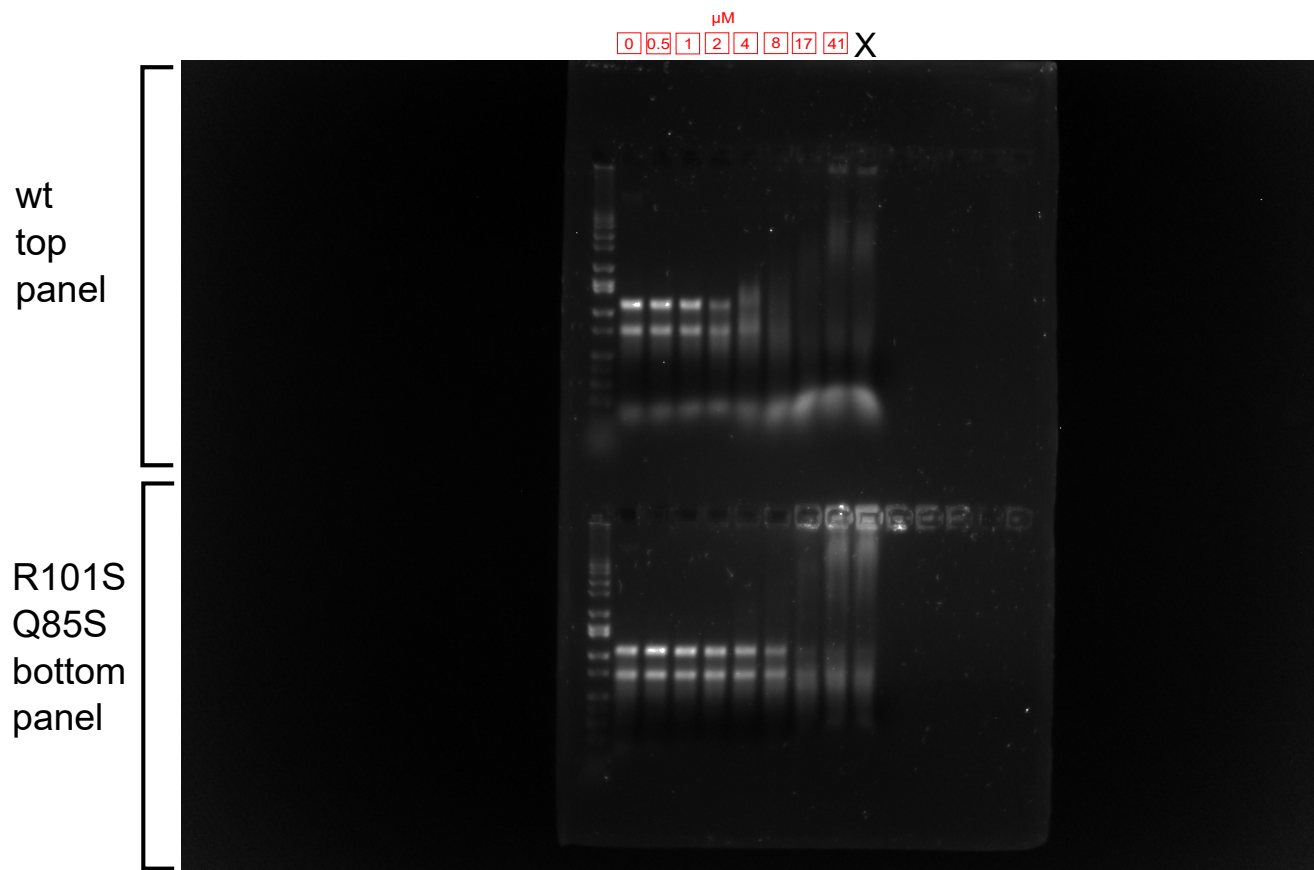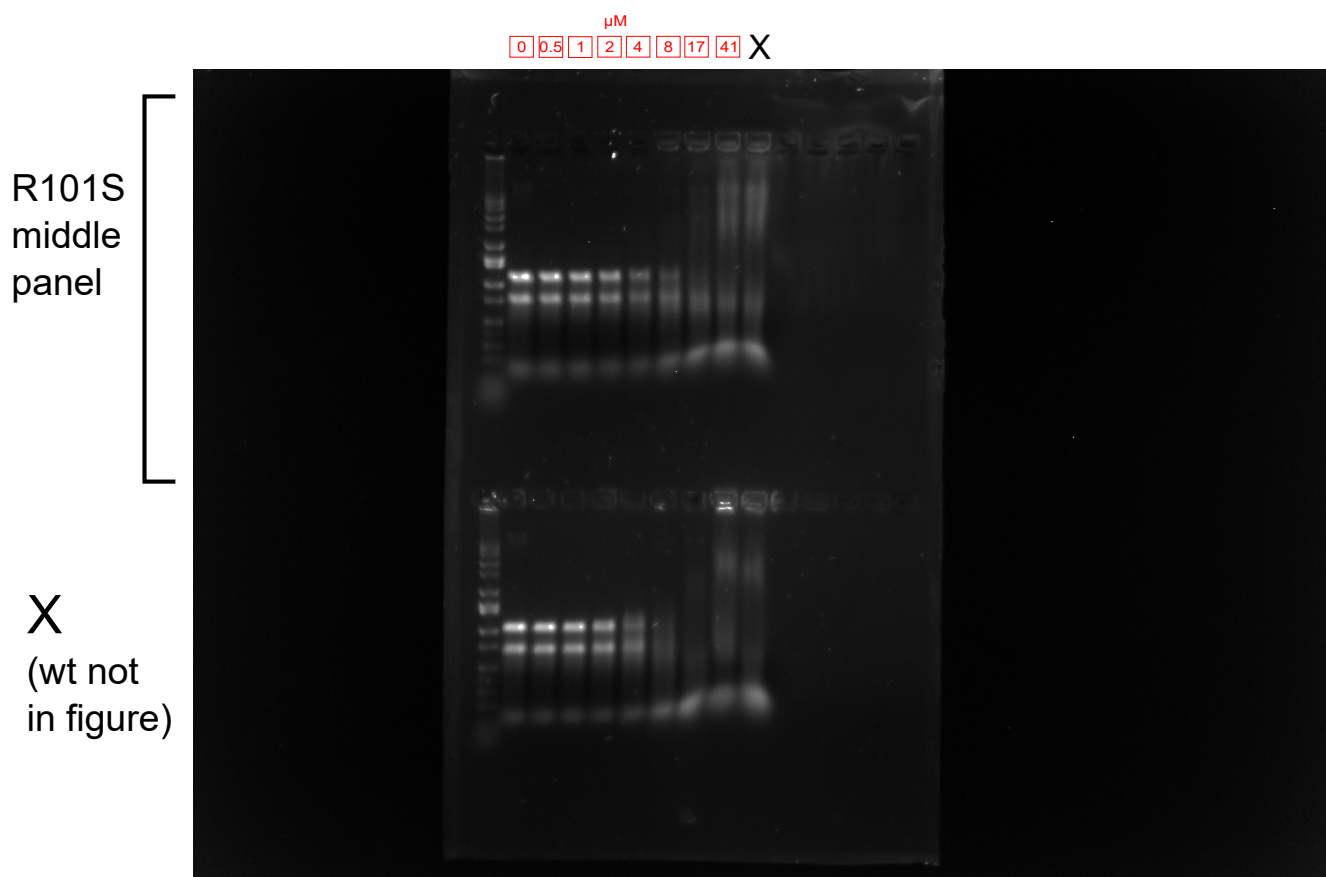

Figure S2 B

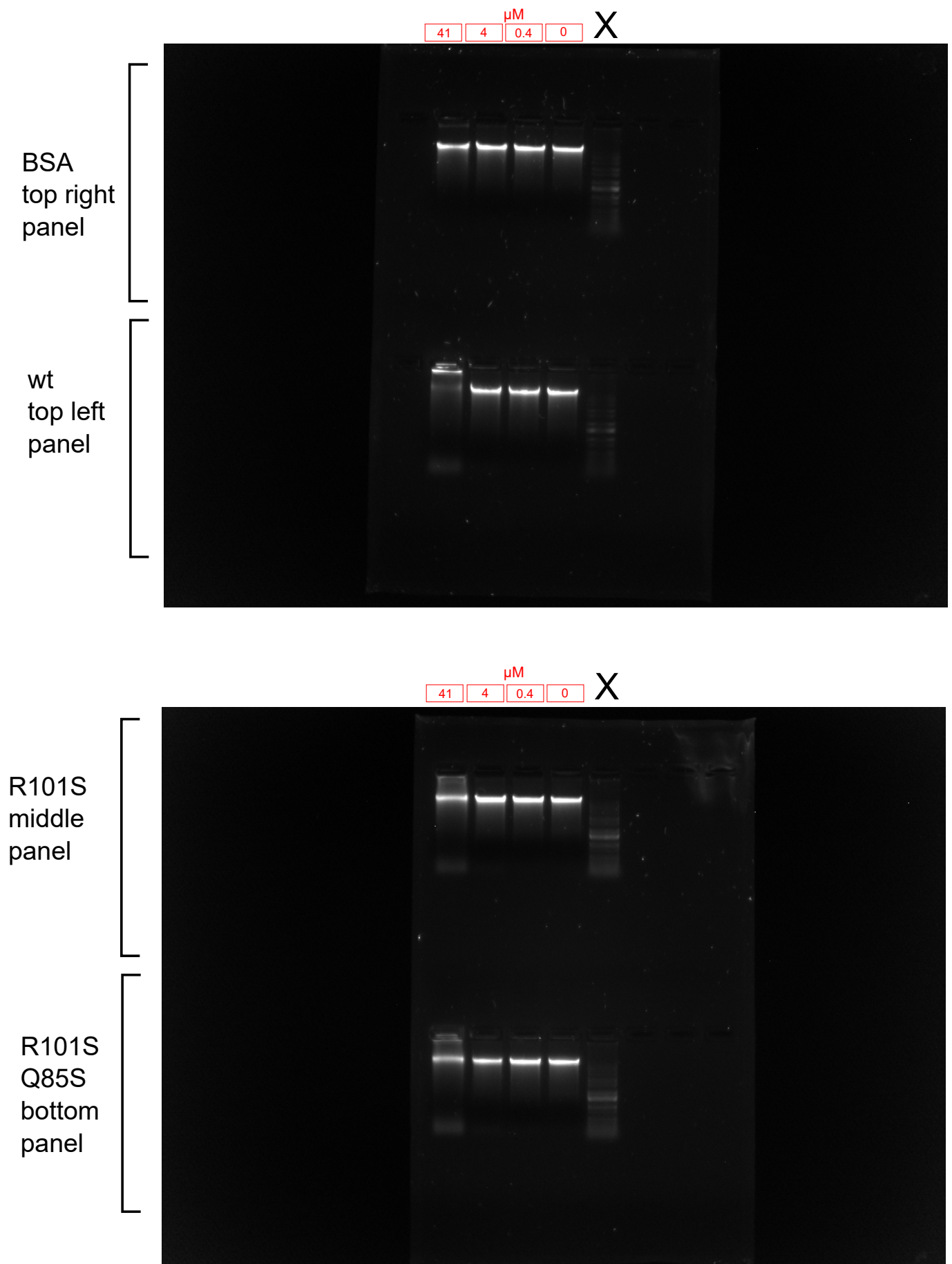

Supplement: S1 Raw images — (PDF) [file pone.0256070.s005.pdf]
